# Peritoneal Infection and Inflammation

**DOI:** 10.1155/2012/456985

**Published:** 2012-06-07

**Authors:** Christoph Aufricht, Wolfgang Neuhofer, Nicholas Topley, Markus Wörnle

**Affiliations:** ^1^Department of Pediatrics and Adolescent Medicine, Medical University of Vienna, Waehringer Gürtel 18-20, 1090 Vienna, Austria; ^2^Department of Physiology, University of Munich, Pettenkoferstraße 12, 80336 Munich, Germany; ^3^Medical Clinic and Policlinic IV, University of Munich, Ziemssenstrasse 1, 80336 Munich, Germany; ^4^Institute of Translation, Innovation, Methodology & Engagement (TIME), Cardiff University School of Medicine, Heath Park, Cardiff CF14 4XN, UK

Peritonitis is a major complication of various disease entities in internal medicine, surgery, and gynecology and, despite treatment advances, remains associated with unacceptably high morbidity and mortality. Causes of peritonitis are manifold including infectious, autoimmune, or chemical processes. A precise knowledge of inflammatory mechanisms in the initiation and establishment of peritoneal inflammation is of pivotal importance for the prevention and treatment of peritonitis. 

This special issue aims to bring the molecular mechanisms of inflammatory processes in the peritoneal cavity into focus, regardless of the organ system or the specific syndrome they result from.

In this special issue of *Mediators of Inflammation*, we are pleased to present to the reader a series of special features written by designated experts in the field.

Local production of proinflammatory mediators at sites of tissue and cell injury within the peritoneal membrane plays a critical role in the propagation of peritoneal inflammation. In addition to infectious causes, various forms of peritoneal damage, induced by surgical trauma and peritoneal dialysis, compromise peritoneal integrity and can ultimately lead to tissue damage, fibrosis, adhesions, or even in the most severe cases the development of encapsulating peritoneal sclerosis. Peritoneal mesothelial cells appear to play a major role in these processes as they are capable of expressing a raft of proinflammatory and immune-modulatory mediators that either directly mediate fibrotic remodelling or attract immune cells to the peritoneal cavity or participate in immune activation. Therapeutic modulation of this sequence of events could provide novel avenues to attenuate peritoneal inflammation and its resulting tissue damage. The present special issue presents novel data on this topic, particularly underscoring the potential impact of the peroxisome proliferator-activated receptor-gamma (PPAR-*γ*) in regulating peritoneal inflammation and fibrosis. Furthermore, a novel PEGylated Toll-like receptor 7 (TLR7) ligand might be of great value for treatment of mast-cell-mediated neutrophilic inflammation.

M. W. J. A. Fieren describes the regulatory role of cytokines, macrophages, and leukocytes during local inflammatory processes in the peritoneal cavity. These processes are of special interest in patients on peritoneal dialysis treatment. A successful peritoneal dialysis treatment requires an intact peritoneal membrane. Treatment can be complicated by infectious or noninfectious sterile peritonitis due to the use of nonphysiologic dialysis solutions. S. Yung and T. M. Chan present an overview of pathophysiological changes to the peritoneal membrane during peritonitis related to peritoneal dialysis. G. Baroni et al. focus on structural changes during peritoneal dialysis-related inflammation. Moreover, experimental data presented in this special issue point to a novel role of the osmosensitive transcription factor NFAT5 in osmolality-induced expression of chemokines in human mesothelial cells. This is of special interest because peritoneal dialysis solutions are usually hypertonic. A rare but fatal complication of peritoneal dialysis treatment is sclerosing peritonitis; in their review article, M. Merkle and M. Wörnle overview causes and treatment options, a complication which can also be seen after kidney or liver transplantation.

Peritoneal adhesions frequently occur after abdominal surgery. Patients with this complication present with abdominal and pelvic pain or intestinal mechanical obstruction. In this special issue, C. Di Filippo et al. provide evidence for a pivotal role of the ubiquitin-proteasome system in the formation of experimental postsurgical peritoneal adhesions. Furthermore, the authors show a possible therapeutic strategy to prevent adhesion formation by blocking the ubiquitin-proteasome system. W. Kessler et al. present novel data on the particular importance of the vagus nerve for the pathophysiology of peritonitis. According to their data, a functioning vagus nerve has a significant modulating influence on mortality in an animal model of polymicrobial sepsis.

In this special issue, T. Kelkka et al. present data in arthritis and peritonitis mice models, where overexpression of SOD3 results in anti-inflammatory effects that are largely independent of NOX2-mediated oxidative bursts. The anti-inflammatory action of extracellular superoxide dismutase (SOD3) is mediated by the dismutation of superoxide into hydrogen peroxide. To analyze whether SOD3 can regulate inflammation in the absence of functional NOX2 complex, T. Kelkka et al. used an elegant design, comparing anti-inflammatory effects of overexpression of SOD3 in Ncf1^−/−^ mice with a deficient oxidative burst with that in wild-type mice. The relevance of these findings will need verification in the clinical setting; however, SOD3 is an important redox regulatory enzyme, and polymorphisms in SOD3 gene are associated with COPD, coronary artery disease, myocardial infarction, and acute lung injury.

We would like to thank all contributors and reviewers for their support to this special issue and feel sure that readers will learn much about this important topic.

*Christoph Aufricht* *Christoph Aufricht* *Wolfgang Neuhofer* *Wolfgang Neuhofer* *Nicholas Topley* *Nicholas Topley* *Markus Wörnle* *Markus Wörnle*

